# Esophageal anthracosis occurred after treatment of esophageal tuberculosis secondary to mediastinal tuberculous lymphadenitis: a rare case report

**DOI:** 10.1186/s12879-023-08095-1

**Published:** 2023-03-21

**Authors:** Weixin Cheng, Xinxin Zhou, Miaomiao Lu, Xi Jin, Feng Ji

**Affiliations:** Department of Gastroenterology, The First Affiliated Hospital, Zhejiang University School of Medicine, Hangzhou, 310003 Zhejiang China

**Keywords:** Gastrointestinal tuberculosis, Lymph node tuberculosis, Anthracosis, Gastrointestinal endoscopy, Endoscopic ultrasound-guided fine needle aspiration, Case report

## Abstract

**Background:**

Anthracosis is a disease generally considered to be in the lungs resulting from exposure to industrial dust in the workplace. Esophageal anthracosis is a fairly rare phenomenon and shows a strong correlation with tuberculosis. Moreover, esophageal involvement in tuberculosis is also rare. We here present an extremely rare case in which follow-up gastroesophageal endoscopy revealed a mass with a sunken, black area in the center and raised ring-like pattern in the surrounding mucosa resembling malignant melanoma. Uncovering the patient’s tuberculosis history finally avoided a misdiagnosis or overtreatment.

**Case presentation:**

A 67-year-old male patient was admitted to the hospital due to “repeated chest pain for 1 month”. Endoscopic ultrasonography and contrast-enhanced CT scans revealed a mass adjacent to the esophageal wall with unclear boundaries. Aspiration biopsy confirmed that esophageal tuberculosis was caused by nearby mediastinal tuberculous lymphadenitis. After a standard anti-tuberculosis treatment regimen, the patient achieved a favorable prognosis. The follow-up gastroesophageal endoscopy showed a sunken black lesion with elevated peripheral mucosa replacing the original tuberculous mass, which was thought to be anthracosis, a disease that rarely occurs in the esophagus.

**Conclusion:**

The diagnosis of tuberculosis should be taken into consideration when a submucosal mass appears in the middle part of the esophagus. Endoscopic ultrasonography can effectively contribute to a definite diagnosis. Moreover, this is the first case of esophageal anthracosis observed only 1 year after the treatment of tuberculosis, indicating esophageal anthracosis can be a short-term disease. The traction of the reduction of tubercular mediastinal lymph nodes after anti-tuberculosis treatment may create a circumstance for pigmentation or dust deposition.

## Background

Anthracosis is a disease generally considered to be in the lungs resulting from exposure to industrial dust in the workplace [1]. In the past few decades, several cases of esophageal anthracosis have been reported in succession. Most of these cases show a strong correlation between anthracosis and tuberculosis [2–6].

As the diagnosis and treatment of pulmonary tuberculosis has become increasingly accurate and effective, awareness of extrapulmonary tuberculosis and multidrug-resistant tuberculosis has started to increase. Endoscopic ultrasonography (EUS) and aspiration biopsy play vital roles in diagnosing esophageal tuberculosis (ET).

In our case, follow-up gastroesophageal endoscopy revealed a mass with a sunken, black area in the center and raised ring-like pattern in the surrounding mucosa, resembling malignant melanoma. Uncovering the patient’s ET history finally avoided a misdiagnosis. This is the first case of esophageal anthracosis observed only 1 year after the treatment of tuberculosis. This study provides a new perspective to explore the mechanism behind anthracosis in the esophagus: the reduction of tubercular mediastinal lymph nodes after anti-tuberculosis treatment (ATT) may create a circumstance for pigmentation or dust deposition.

## Case presentation

A 67-year-old male patient was admitted to the hospital due to “repeated chest pain for 1 month” in April 2018. The pain was behind the sternum, paroxysmal, and often occurred when waking up. The pain was tolerable, and could be relieved after eating meals. He also had sour regurgitation at that time, without dysphagia, nausea, vomiting, fever, chills or any other discomfort. No obvious weight loss was observed.

The patient had a history of *H. pylori* infection and was treated with levofloxacin, clarithromycin, colloidal bismuth pectin and omeprazole. He also had a history of chronic obstructive pulmonary disease for 5 years. He started smoking and drinking 40 years ago, consuming 450 mL beer and smoking 10 cigarettes per day, but he gave up these habits 20 years ago. No allergy of food or drugs.

Vital signs: T: 37.1 °C. P: 90/min. R: 18/min. BP: 122/81 mmHg.

**Fig. 1:**
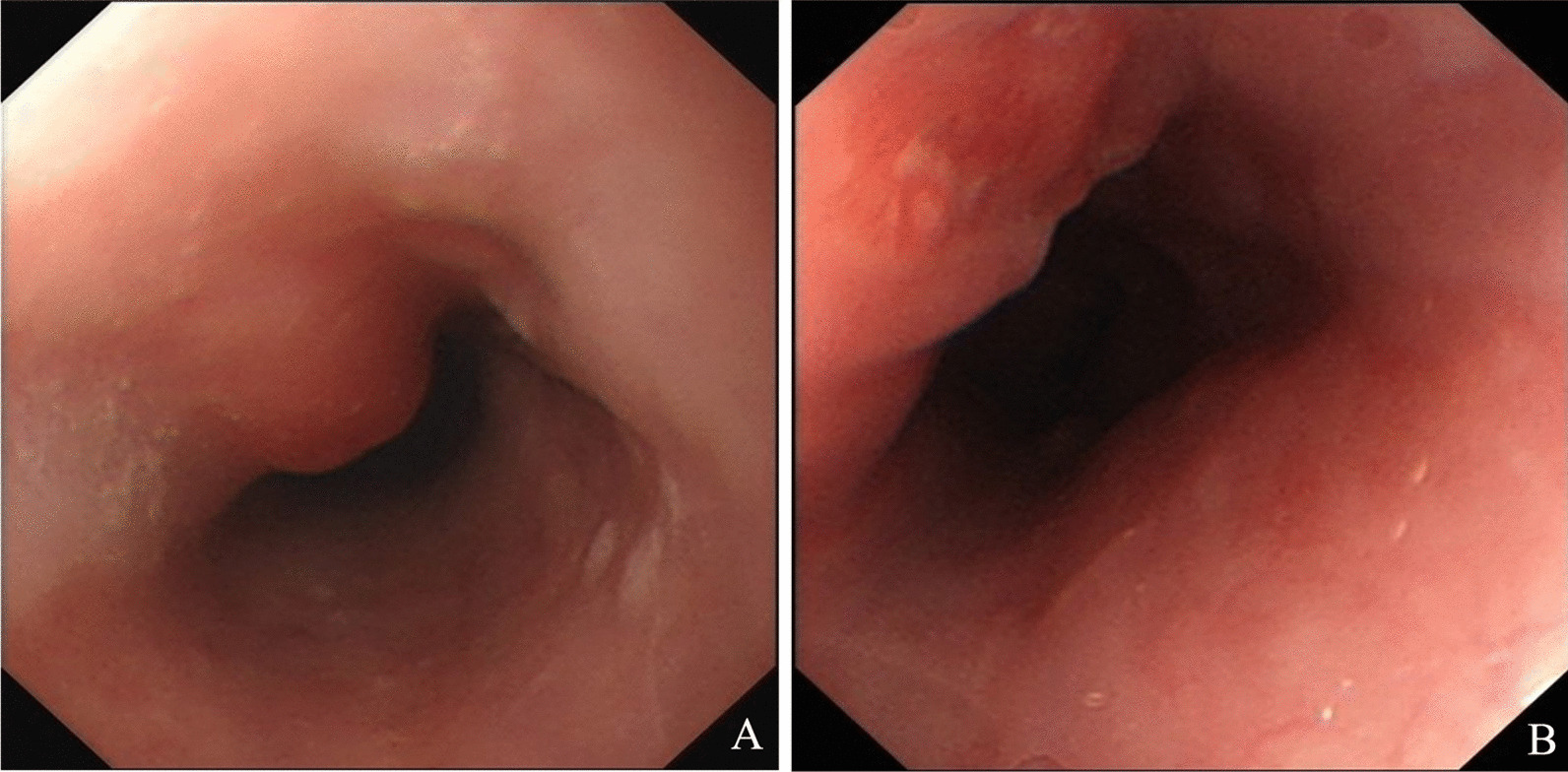
Gastroesophageal endoscopic result showed significant changes within 3 months. **A** First gastroesophageal endoscopy showed a smooth-surface submucosal mass, at 25 cm from the incisors in esophagus. **B** 3 months later the endoscopy showed a protuberance with rough surface, at 25 cm from the incisors in esophagus

**Fig. 2:**
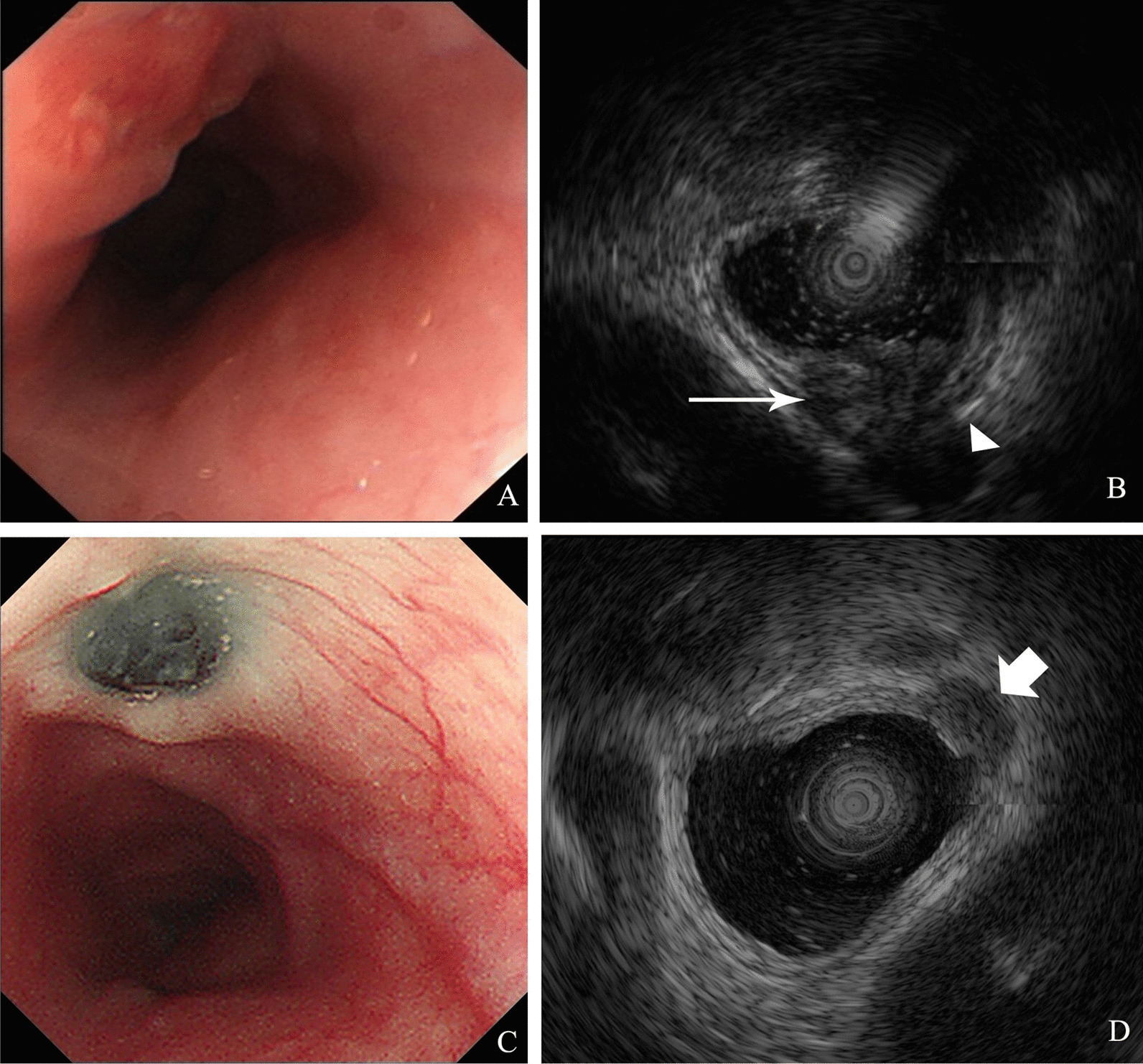
Endoscopy with EUS results before and after anti-tuberculosis treatment. **A** Endoscopy showed a protuberance with rough surface before ATT. **B** EUS revealed the layer structure of esophageal wall disappeared over the protuberance, instead of which was a hypoechoic structure (long arrow) spreading to the adventitia. A round-like hypoechoic nodule (arrowhead) could be seen at the back, and the boundary between it and the esophageal mass was not clear. **C** After ATT the follow-up gastroesophageal endoscopy showed a black sunken lesion with elevated peripheral mucosa replaced the original tuberculous mass. **D** EUS revealed a hypoechoic nodular structure (short arrow), where the layers of esophageal wall were disappeared. The measured size of mass was smaller than (**B**)

**Fig. 3:**
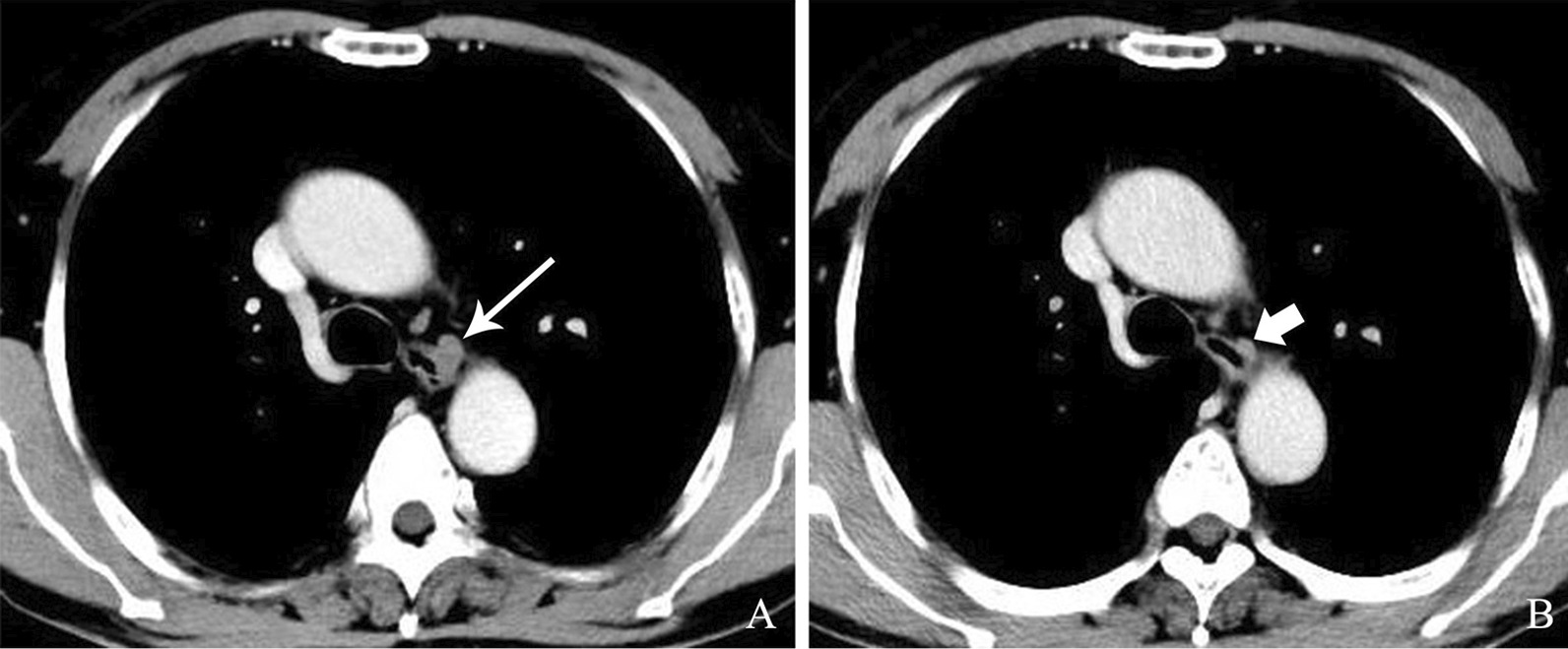
Contrast enhancement CT scan before and after anti-tuberculosis treatment. **A** Contrast enhancement CT scan before ATT showed a homogenous density mass (long arrow) adjacent to esophageal wall. **B** After ATT, follow-up contrast enhancement CT scan showed an obviously shrunk mass (short arrow)

Three months ago (January 2018), gastroesophageal endoscopy showed a submucosal mass with a smooth surface 25 cm from the incisors in the esophagus (Fig. 1A). At this time, the patient came to the hospital because of chest pain, so another gastroesophageal endoscopy (April 2018) was performed. Endoscopy showed a protuberance with a rough surface 25 cm from the incisors in the esophagus (Fig. 1B). EUS revealed that the layered structure of the esophageal wall disappeared over the protuberance, instead showing a hypoechoic structure spreading to the adventitia. A circular hypoechoic nodule could be seen at the back of the esophagus, and the boundary between the nodule and the esophageal mass was not clear. The measured thickness at the thickest part was 1.40 cm. The partial section size was 2.52 * 1.82 cm (Fig. 2B). A contrast-enhanced CT scan showed a mass with homogenous density adjacent to the esophageal wall (Fig. 3A). In addition, TSPOT test (April 2018) showed the positive result.

In view of our patient’s first endoscopic result, the first impression was submucosal tumors (SMTs). The patient was coming for treating sour regurgitation, so he refused to adopt a further diagnostic procedure. He was given rabeprazole for acid suppression, and rebamipide for gastric protection. However, 3 months later, the endoscopic result changed, calling attention to the possibility of tuberculosis. At the same time, a mass adjacent to the esophageal wall with homogenous density was seen on CT, which was considered to be enlarged lymph nodes.

The typical EUS manifestation of ET is the destruction of the five-layer esophageal wall structure; as a result, all layers are involved or only the mucosal layer is intact. Heterogeneous or homogeneous hypoechoic masses with mediastinal lymphadenopathy can be convincing proof of ET. Although a malignant lesion in the esophagus infiltrating the mediastinal lymph nodes can also be shown as a full-thickness segment with a hypoechoic structure and enlarged lymph nodes, there are still two differences on EUS between malignancy and ET: (1) malignant lymph nodes do not fuse with the esophageal wall, leading to disruption of the adventitia, and (2) hyperechoic strands and foci are not found in esophageal carcinoma or metastatic lymph nodes [7]. Therefore, we inferred that the lesion was more likely to be a tuberculous mass on the basis of the fusion of the lymph nodes and esophageal wall. The hypothesis was that the enlarged lymph nodes in the mediastinum resulted from mediastinal lymphadenitis infiltrating the esophagus from the serosa to all layers of the esophageal wall, initially suppressing the esophagus so that it appeared as a submucosal bulge, which later progressed to an ulceration.

**Fig. 4:**
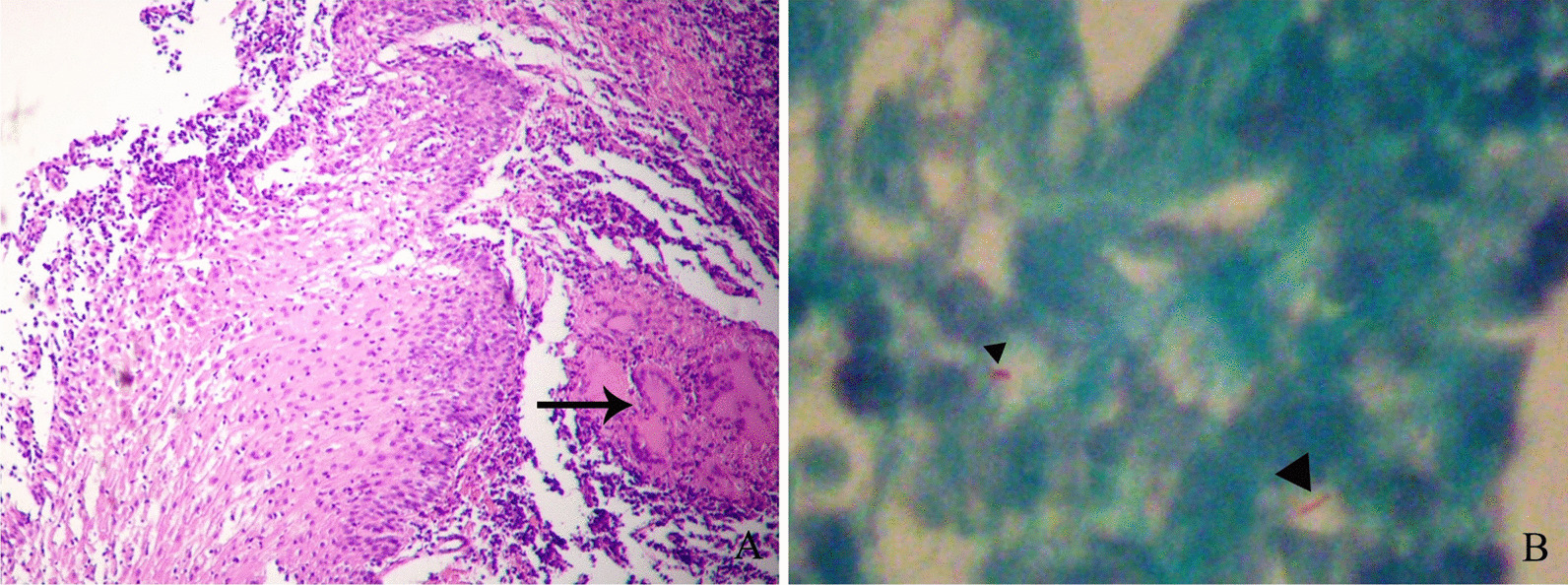
Hematoxylin–eosin and acid-fast staining of the biopsy specimen. **A** Hematoxylin–eosin staining revealed chronic active mucositis with multinuclear giant cell reaction (arrow) in middle of the esophagus. **B** Several acid-fast bacilli (arrowheads) were found in the biopsy specimen with acid-fast staining

EUS-guided fine-needle aspiration (EUS-FNA) was further performed, and the pathological result indicated chronic active mucositis with multinuclear giant cell reaction in the middle of the esophagus (Fig. 4A). More importantly, several acid-fast bacilli were found in the biopsy specimens by acid-fast staining (Fig. 4B).

## Final diagnosis

The final diagnosis in the presented case was esophageal tuberculosis secondary to mediastinal tuberculous lymphadenitis.

## Treatment

Our patient was diagnosed as secondary esophageal tuberculosis, strongly indicating the undiscovered past tuberculosis history of the patient. Thus, according to the guideline for tuberculosis, we initially adopted the following first-line ATT regimen to treat the patient: isoniazid, rifampicin, pyrazinamide and ethambutol for 3 months. During the 3-month ATT, the patient responded well and his chest pain was relieved. He had his routine check-up at local hospital and found no adverse effects on liver function or hematological system. He was then asked to take isoniazid and rifampicin for another 6 months for continuation phase. A definite diagnosis of tuberculosis helped to avoid surgery. The definitive diagnosis also improved the patient’s experience and reduced medical resource consumption.

## Outcome and follow-up

After 1 year, the patient came to the hospital for a routine check-up. His symptoms of chest pain and sour regurgitation disappeared. The follow-up gastroesophageal endoscopy showed a sunken black lesion with elevated peripheral mucosa replacing the original tuberculous mass (Fig. 2C). EUS revealed that the lesion was a hypoechoic nodular structure, and the layers of the esophageal wall disappeared. The measured section size was approximately 0.84 * 0.54 cm, obviously smaller than before (Fig. 2D). Contrast-enhanced CT scans also showed a shrunken mass compared with that on the prior scan (Fig. 3B). Apparently, the diagnosis of ET was correct, and ATT was an effective cure for tuberculosis, but the appearance of a black mass in the esophagus has generated a new issue.

## Discussion

Anthracosis is a disease that generally appears in the lungs, resulting from exposure to industrial dust in the workplace. In addition, anthracosis has also been reported in the liver [8], pleura [9, 10], and lymph nodes [11]. The first case of esophageal anthracosis was reported by Vakharia et al. in 1990 [12]. In the past few decades, several cases of esophageal anthracosis have been reported in succession, although it remains a rare phenomenon; most of these cases show a strong correlation between anthracosis and tuberculosis [2–6].

Typically, tuberculosis affects the lungs (pulmonary TB), but it can also affect other organs (extrapulmonary TB), such as the pleura and lymph nodes [13]. Esophageal involvement in tuberculosis is extremely rare, with an incidence of 0.14–0.15% in all TB patients [14, 15].

There are two types of ET in view of the tubercular involvement of different sites in the body. Primary esophageal tuberculosis indicates that tuberculosis only affects the esophagus, while secondary esophageal tuberculosis is more common and most frequently secondary to hilar or/and mediastinal tuberculous lymphadenitis, sometimes to Potts spine or pulmonary tuberculosis [7, 16–19]. On the other hand, the area mainly involved in ET is the middle part of the esophagus, which may result from the fact that this part is adjacent to the lymph node-rich area under the carina [7, 16].

EUS currently plays a key role in guiding mediastinal lymph node fine-needle cytology and diagnosing ET with outstanding sensitivity and specificity. Moreover, EUS-FNA helps in obtaining a definitive diagnosis and distinguishing ET from other diseases [7, 16–19]. It was rather fortunate that multinuclear giant cell reactions with several acid-fast bacilli were observed in the biopsy specimens in our case, providing a decisive clue for the diagnosis of ET. In fact, the positive rate of acid-fast staining of specimens captured by EUS-FNA is not high, at approximately 37.5–42.4% in ET patients [16–19]. If acid-fast bacilli cannot be found in the biopsy specimens, other histopathological examinations are required to confirm the diagnosis. Furthermore, the gold standard for diagnosing tuberculosis is cultural positive of *Mycobacterium tuberculosis* (MTB) or MTB detected by molecular probe, because nontuberculous mycobacterium is also presented as acid-fast staining positive. Unfortunately, we didn’t do MTB culture to further differentiate ET from esophageal nontuberculous mycobacterium infection. Standard anti-tuberculosis treatment can achieve satisfactory effects in most ET cases. If tuberculosis is highly suspected without evidence of a definite diagnosis, experimental ATT can be applied to observe the reaction. In our case, the positive acid-fast staining result, TSPOT positive result and the excellent therapeutic effect of ATT together contributed to the definite diagnosis of ET.

The difficulties in diagnosing and choosing appropriate treatment for ET may be due to its nonspecific clinical, laboratory, and imaging features [20–23]. The majority of ET patients, as well as the patient in our case, come to the hospital with complaints about dysphagia and/or chest pain. Few patients have typical tubercular symptoms, such as low fever, fatigue and weight loss [18, 19]. Uncommon presentations, such as hematemesis [24], perforation [25] and fistula-related symptoms [26], can also be found in previous studies. Endoscopic presentations of ET are variable, including ulceration, protruding lesions and fistulae, causing ET to be easily misdiagnosed as esophageal carcinoma or SMTs. CT often shows a thickened esophageal wall with enlarged subcarinal lymph nodes. The manifestation of nodes with central low-density areas and peripheral enhancement and findings of homogeneous and calcified nodes are capable of differentiating tuberculous lymphadenitis from other causes of mediastinal lymphadenopathy [18]. Although we didn’t apply needle biopsy to the enlarged mediastinal lymph nodes in this case, the typical CT presentation, typical correlation with ET, and reduced lymph node size following ATT confirmed our diagnosis of mediastinal tuberculous lymphadenitis.

After a standard ATT regimen, a sunken black lesion with elevated peripheral mucosa replaced the original tuberculous mass. Only a few studies have reported similar conditions, according to which we inferred that this was a case of esophageal anthracosis [1, 3–6]. Macroscopically, anthracosis lesions usually appear with a 5 mm or larger circular black area and a central depression, which is similar to the findings in our case. Microscopically, anthracosis lesions are found to be formed of dust-laden macrophages embedded in a network of reticulin fibers [12].

To clarify the differences between esophageal anthracosis and primary malignant melanoma (PMME) is extremely important for clinicians to avoid overtreatment. The endoscopic appearance of most PMME showed polypoid lesions, while anthracosis showed rather flat lesions [1–6]. Immunohistochemical staining can differentiate PMME from anthracosis by positive staining for S-100 protein and HMB-45.

The association of tuberculosis with pulmonary anthracosis has already been confirmed by many studies. Choi, Park, Yang and Nishiyama et al. reported cases of esophageal anthracosis associated with tuberculosis [3–6]. Notably, no other causes of esophageal inflammation, such as Crohn’s disease, have been reported with anthracosis. Periesophageal inflammation, such as lymphadenitis, can also cause adjacent esophageal traction diverticula and dust deposits at the retraction site of the esophageal mucosa [1]. Considering the ET history of the patient and the typical endoscopic manifestation, we diagnosed the patient as esophageal anthracosis without histological examination for the black lesion.

Most of the previous reports evaluated the value of a tuberculosis diagnosis when anthracosis was found in the esophagus or emphasized the importance of differentiating anthracosis from PMME [1, 4, 6]. In our case, anthracosis occurred after the treatment of tuberculosis, which provides a new perspective for exploring the mechanism underlying the relationship between tuberculosis and anthracosis. There are no specific explanations for this relationship in previous studies. As it has been reported that chronic inflammation in the esophagus can result in the abnormal proliferation of melanocytes, the factor of endogenous melanin production should be taken into consideration [27]. Other studies demonstrated the black appearance of anthracosis were related to circumstances surrounding smoke and powder or personal habits such as smoking and drinking [2, 28]. The patient in our case has been living in countryside and has a history of smoking. The plant ashes from straw burning and smoke might contribute to the dust deposition in the lesion. Thus, the anthracosis observed in our case was possibly caused by esophageal traction diverticula due to the reduced size of tubercular mediastinal lymph nodes after ATT, with endogenous melanosis resulting from chronic mucosa-stimulating conditions or exogenous dust-like substances (such as smoke or gas fumes) depositing in the lesion.

Akazaki and Inagaki in Japan reported that dust-disposing cells can play crucial roles not only in the process of depositing dust in the lung but also in the fight against invading tubercle bacilli, and the appearance of both pneumoconiotic and tuberculous lesions in the same location seems to accelerate the aggravation of tuberculous complications [26]. This may imply the possibility that the dust-disposing cells aggregate to defend against tuberculosis. The underlying mechanism still needs investigation.

## Conclusion

As the diagnosis and treatment of pulmonary tuberculosis have become increasingly accurate and effective, awareness of extrapulmonary tuberculosis and multidrug-resistant tuberculosis has started to increase. This study aimed to report that the diagnosis of tuberculosis should be taken into consideration when a submucosal mass appears in the middle part of the esophagus and emphasize the importance of EUS or EUS-FNA in diagnosing esophageal tuberculosis. Although a few articles have reported that esophageal anthracosis could be a sentinel disease of tuberculosis and only one study has reported a patient with esophageal anthracosis and a prior history of tuberculosis 20 years ago, no study has shown anthracosis that occurred a short period after the treatment of tuberculosis [10]. Our case indicates anthracosis can rapidly develop, as traction of the reduced size of tubercular mediastinal lymph nodes after ATT may create a circumstance for pigmentation or dust deposition.

## Data Availability

The datasets used during the study are available from the corresponding authors on reasonable request. The identifying patient data are unavailable on account of protecting patients’ privacy.

## Abbreviations

EUS: Endoscopic ultrasonography
ET: Esophageal tuberculosis
ATT: Anti-tuberculosis treatment
SMTs: Submucosal tumors
EUS-FNA: EUS-guided fine-needle aspiration
MTB: *Mycobacterium tuberculosis*
PMME: Primary malignant melanoma
